# Supplementary material for: Explicit description of viral capsid subunit shapes by unfolding dihedrons
Source: Commun Biol. 2024 Nov 14;7:1509. doi: 10.1038/s42003-024-07218-x (PMC11564659; doi:10.1038/s42003-024-07218-x)
Supplement: Supplementary file 3 — Description of Additional Supplementary File [file 42003_2024_7218_MOESM3_ESM.pdf]

Description of additional supplementary file

File name: Supplementary Movie 1.

Description: Unfolding of a dihedron when the junction point is inside the triangle.

File name: Supplementary Movie 2.

Description: Unfolding of a dihedron when the junction point is outside the triangle.

File name: Supplementary Movie 3.

Description: Unfolding of a dihedron. Cutting along a complex curve.
